# Attitudes Toward COVID-19 Vaccines Among Pregnant and Recently Pregnant Individuals

**DOI:** 10.1001/jamanetworkopen.2024.5479

**Published:** 2024-04-08

**Authors:** Joshua T. B. Williams, Kate Kurlandsky, Kristin Breslin, M. Joshua Durfee, Amy Stein, Laura Hurley, Jo Ann Shoup, Liza M. Reifler, Matthew F. Daley, Bruno J. Lewin, Kristin Goddard, Michelle L. Henninger, Jennifer C. Nelson, Gabriela Vazquez-Benitez, Kayla E. Hanson, Candace C. Fuller, Eric S. Weintraub, Michael M. McNeil, Simon J. Hambidge

**Affiliations:** 1 Ambulatory Care Services, Denver Health and Hospitals, Denver, Colorado; 2 Department of Pediatrics, University of Colorado School of Medicine, Aurora; 3 Institute for Health Research, Kaiser Permanente Colorado, Aurora; 4 Kaiser Permanente Southern California, Pasadena; 5 Kaiser Permanente Vaccine Study Center, Oakland, California; 6 Center for Health Research, Kaiser Permanente Northwest, Portland, Oregon; 7 Health Research Institute, Kaiser Permanente Washington, Seattle; 8 HealthPartners Institute, Minneapolis, Minnesota; 9 Marshfield Clinic Research Institute, Marshfield, Wisconsin; 10 Department of Population Medicine, Harvard Pilgrim Health Care Institute, Boston, Massachusetts; 11 Immunization Safety Office, US Centers for Disease Control and Prevention, Atlanta, Georgia

## Abstract

**Importance:**

Pregnant people and infants are at high risk of severe COVID-19 outcomes. Understanding changes in attitudes toward COVID-19 vaccines among pregnant and recently pregnant people is important for public health messaging.

**Objective:**

To assess attitudinal trends regarding COVID-19 vaccines by (1) vaccination status and (2) race, ethnicity, and language among samples of pregnant and recently pregnant Vaccine Safety Datalink (VSD) members from 2021 to 2023.

**Design, Setting, and Participants:**

This cross-sectional surveye study included pregnant or recently pregnant members of the VSD, a collaboration of 13 health care systems and the US Centers for Disease Control and Prevention. Unvaccinated, non-Hispanic Black, and Spanish-speaking members were oversampled. Wave 1 took place from October 2021 to February 2022, and wave 2 took place from November 2022 to February 2023. Data were analyzed from May 2022 to September 2023.

**Exposures:**

Self-reported or electronic health record (EHR)–derived race, ethnicity, and preferred language.

**Main Outcomes and Measures:**

Self-reported vaccination status and attitudes toward monovalent (wave 1) or bivalent Omicron booster (wave 2) COVID-19 vaccines. Sample- and response-weighted analyses assessed attitudes by vaccination status and 3 race, ethnicity, and language groupings of interest.

**Results:**

There were 1227 respondents; all identified as female, the mean (SD) age was 31.7 (5.6) years, 356 (29.0%) identified as Black race, 555 (45.2%) identified as Hispanic ethnicity, and 445 (36.3%) preferred the Spanish language. Response rates were 43.5% for wave 1 (652 of 1500 individuals sampled) and 39.5% for wave 2 (575 of 1456 individuals sampled). Respondents were more likely than nonrespondents to be White, non-Hispanic, and vaccinated per EHR. Overall, 76.8% (95% CI, 71.5%-82.2%) reported 1 or more COVID-19 vaccinations; Spanish-speaking Hispanic respondents had the highest weighted proportion of respondents with 1 or more vaccination. Weighted estimates of somewhat or strongly agreeing that COVID-19 vaccines are safe decreased from wave 1 to 2 for respondents who reported 1 or more vaccinations (76% vs 50%; χ^2^_1_ = 7.8; *P* < .001), non-Hispanic White respondents (72% vs 43%; χ^2^_1_ = 5.4; *P* = .02), and Spanish-speaking Hispanic respondents (76% vs 53%; χ^2^_1_ = 22.8; *P* = .002).

**Conclusions and Relevance:**

Decreasing confidence in COVID-19 vaccine safety in a large, diverse pregnant and recently pregnant insured population is a public health concern.

## Key Points

**Question:**

Do perceptions of COVID-19 vaccines differ among distinct pregnant or recently pregnant respondents from 2 survey waves between November 2021 and February 2023?

**Findings:**

In this survey study, 1227 of 2956 people responded. Respondents who received 1 or more COVID-19 vaccines, identified as non-Hispanic White, and preferred the Spanish language had the largest decreases in agreement that COVID-19 vaccines are safe across waves.

**Meaning:**

These findings suggest that decreases in perceived COVID-19 vaccine safety among specific groups of insured pregnant and recently pregnant individuals is a public health concern.

## Teaser

This survey study evaluates changing attitudes regarding COVID-19 vaccines by vaccination status and race, ethnicity, and language.

## Introduction

Pregnant people infected with SARS-CoV-2 virus have increased risks of morbidity, mortality, and adverse pregnancy outcomes.^1,2,3,4,5,6^ While pregnant people were not initially included in COVID-19 vaccination trials, observational data suggest vaccination during pregnancy reduces the risks of severe COVID-19 disease and adverse birth outcomes.^7,8,9,10,11,12,13^ Young children are also at risk of severe disease and death from COVID-19, with infants under 6 months old with the highest rates of severe COVID-19 outcomes.^14,15^ Early pandemic studies found consistently low levels of vaccination intention or uptake among pregnant people, with low rates in Black and Latino individuals.^16,17,18,19,20^

As the COVID-19 public health emergency ended, these trends continued. As of July 29, 2023, Vaccine Safety Datalink (VSD) surveillance found just 16.2% of pregnant people aged 18 to 49 years had received a COVID-19 booster vaccine, with only 8.3% of Black pregnant people and 9.6% of Latino pregnant people vaccinated during pregnancy.^21^ Assessing attitudes toward COVID-19 vaccines among pregnant and recently pregnant people is critical to public health messaging and clinician counseling.^22^ We conducted 2 cross-sectional surveys of distinct samples of pregnant and recently pregnant individuals during the latter portion of the COVID-19 pandemic. Our primary aims were to assess trends in attitudes regarding COVID-19 vaccines by (1) self-reported vaccination status and (2) race, ethnicity, and preferred language.

## Methods

### Overview

The Colorado Multiple Institutional Review Board (COMIRB) approved this survey study; participating sites’ institutional review boards ceded oversight to COMIRB. COMIRB granted a waiver of written consent for study participation because the survey’s introduction language made it clear a respondent’s choice to complete the survey indicated consent to participate. This work was part of a larger project assessing vaccination attitudes and status among pregnant and nonpregnant VSD members over time.^21^ Distinct cohorts of pregnant and recently pregnant members were sampled over time, with the first survey administered from November 1, 2021, to February 1, 2022, and the second survey administered from October 1, 2022, to February 1, 2023. Respondents were asked to report their COVID-19 vaccination status and share their attitudes toward COVID-19 infection and monovalent COVID-19 vaccines (wave 1) or bivalent Omicron booster vaccines (wave 2). Self-reported vaccination status was considered the criterion standard, as with prior attitudinal surveys in the VSD.^23^ We followed the American Association for Public Opinion Research (AAPOR) reporting guideline for survey studies, applying Response Rate Definition 6.^24^

### Setting

The VSD is a collaboration between the US Centers for Disease Control and Prevention (CDC) and 13 integrated health care systems (called sites).^25^ Vaccination data were derived from the electronic health record (EHR) and reconciled routinely with state immunization information systems.^26^ VSD members represent over 3% of the total US population.^27^ Eight VSD sites contributed data: Denver Health (Colorado), Kaiser Permanente Colorado, Marshfield Clinic (Wisconsin), HealthPartners (Minnesota), Kaiser Permanente Washington, Kaiser Permanente Northwest (Oregon), Kaiser Permanente Northern California, and Kaiser Permanente Southern California.

### Study Participants: Identification of Pregnant Persons

The VSD has a validated algorithm to identify pregnancies in near real-time with a high degree of accuracy.^28,29,30^ We used this algorithm to identify adults aged 18 to 49 years who were pregnant any time between December 11, 2020, and August 31, 2021 (wave 1), or January 1, 2022, and August 1, 2022 (wave 2). As time elapsed between sampling and surveys going into the field, respondents included currently and recently pregnant people. Eligible individuals had continuous health insurance enrollment during the same periods for each wave, except at Denver Health, which uses empanelment as a proxy for enrollment.^31^ We excluded people with an *International Statistical Classification of Diseases, Tenth Revision, Clinical Modification* code for adverse pregnancy outcomes (eg, spontaneous abortion, anencephaly), those with possible data errors (eg, simultaneous administration of multiple COVID-19 vaccines), and individuals who had opted out of research.

### Study Participants: Sampling Procedures

Within eligible cohorts, we conducted stratified sampling at each VSD site using 6 mutually exclusive strata defined by the following EHR factors: COVID-19 vaccination (unvaccinated or ≥1 vaccines), race (Black or other race and ethnicity, which included American Indian or Alaskan Native, Asian, Native Hawaiian or Pacific Islander, and White), and preferred language (English or Spanish). We oversampled individuals roughly 2:1 whose EHR indicated they were unvaccinated or self-identified as Black race or preferred Spanish language, as prior surveys of pregnant VSD members have shown lower response rates in these groups.^23^ Spanish-speaking participants were sampled from Denver Health and Kaiser Permanente Southern California.^32^ Separate samples were created for each wave.

### Sample Size

The target sample size for each survey wave was determined by a related project outcome: the accuracy of EHR vaccination data vs self-reported vaccination. Assuming variable response rates by vaccination status and race,^23^ a 60% negative predictive value (EHR-unvaccinated people reporting they were unvaccinated), and a sample of 907 unvaccinated individuals and 593 vaccinated individuals for each survey wave, the study was powered to achieve a 2.0% CI around the vaccination confirmation rate and an 8.0% CI around the nonvaccination confirmation rate in each wave. Estimated CIs were corrected for the anticipated response rate.

### Survey Design and Cognitive Interviews

Survey instruments aligned with the Health Belief Model.^33,34,35,36^ We based our survey questions on previously published instruments,^23,37,38,39^ using wording from COVID-19 studies when possible.^40,41,42,43^ One question probed perceptions of trusted information sources, and as response categories changed between waves, we have presented data for this question from wave 2 only. We included questions about race, ethnicity, household income, household size, and highest educational attainment. For respondents, self-reported race and ethnicity were the reference standards^44^; EHR data were used when survey data were missing. Race was assessed in this study because vaccination rates have been found to be lower for Black and Latino individuals.^16,17,18,19,20^

Draft surveys underwent a first round of revisions from VSD site content experts. Afterwards, the survey was translated into Spanish by a certified bilingual translator. It was pilot tested at Denver Health with 20 pregnant people (10 in English, 10 in Spanish). Interviewees were recruited from pediatric and obstetric waiting rooms, expressed verbal consent, and received a $25 gift card. A second round of revisions incorporated interviewees’ feedback for English and Spanish versions. Surveys were reviewed a third time by VSD sites and CDC experts (J.T.B.W., K.K., K.B., L.H., J.A.S., L.M.R., M.F.D., B.J.L., K.G., M.L.H., J.C.N., G.V.B., K.E.H., C.C.F., E.S.W., M.M.M., and S.J.H.) and finalized.

Due to the timing of survey administration and official vaccine recommendations, question wording regarding attitudes toward COVID-19 vaccines changed between waves. In wave 1, questions about COVID-19 vaccines referred generally to “COVID-19 vaccines” (ie, original monovalent mRNA and adenoviral vector vaccines). In wave 2, questions referred specifically to the “COVID-19 Omicron booster vaccines,” and question instructions noted that these vaccines were also called “bivalent booster vaccines.” Questions about COVID-19 booster vaccines also differed. In wave 1, we asked, “Have you received one or more COVID-19 booster vaccines?” In wave 2, we asked, “Have you received a COVID-19 Omicron booster vaccine?” The Figure illustrates changes in recommendations over time.^45,46^

**Figure.**
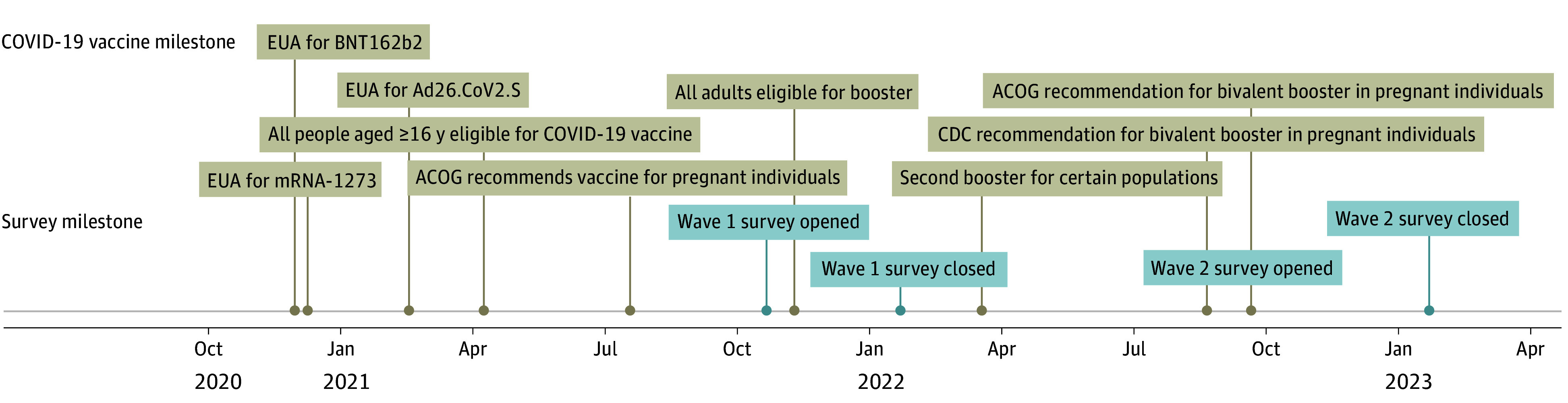
Timeline of Survey Waves 1 and 2 in Relation to the Centers for Disease Control and Prevention (CDC) and American College of Obstetricians and Gynecologists (ACOG) COVID-19 Vaccine Emergency Use Authorizations (EUAs) and Subsequent Recommendations for Monovalent and Bivalent (Omicron) Booster COVID-19 Vaccines in Pregnant Individuals Recommendations directly from CDC; regarding bivalent (Omicron) booster, see CDC, 2022.^45^ Recommendations directly from ACOG; regarding bivalent (Omicron) booster, see ACOG, 2022.^46^

### Survey Administration

Prospective participants received up to 10 survey invitations via postal mail, e-mail, and telephone calls, per tailored survey design best practices.^47^ Survey administration was consistent across VSD sites, except 1 site required participants to receive a presurvey letter with an opportunity to opt out and prohibited contact by email or phone. Outreach stopped after survey completion or if a person opted out. Surveys were hosted online via Research Electronic Data Capture Software.^48^ Respondents received a $25 gift card. We obtained a waiver of written consent, but participants could opt out by email, in writing, or by phone.

### Statistical Analysis

We defined respondents as people who completed the first survey question: “Have you received a COVID-19 vaccine?” We used the Pearson χ^2^ test to compare survey respondents to nonrespondents on sociodemographic variables available via EHR using 2-sided tests, considering a *P* value less than .05 significant. Among respondents, we calculated weighted descriptive statistics for any self-reported COVID-19 vaccination (ie, ≥1 dose ever), self-reported monovalent booster vaccination among those who had received at least 1 prior COVID-19 vaccine (wave 1), and self-reported Omicron booster vaccination among those who had received at least 1 prior COVID-19 vaccine (wave 2). For attitudinal and sociodemographic measures, we compared respondents by self-reported vaccination status (unvaccinated vs receipt of ≥1 dose) and by 3 mutually exclusive race, ethnicity, and language groups (non-Hispanic White, non-Hispanic Black, and Spanish-speaking Hispanic of any race).

Missingness in the dataset was low (<10%), and unknown or missing categories were included in analyses. We used the Rao-Scott χ^2^ test for weighted tables using 2-sided hypothesis testing, considering a *P* value less than .05 significant. We included a finite population correction and incorporated inverse probability weighting to account for sampling and response probability by VSD site, vaccination status, and oversampling of non-Hispanic Black and Spanish-speaking Hispanic people. When considering both waves, individual survey weights were adjusted to reflect an average annual population.^49^ Analyses were conducted using SAS version 9.4 (SAS Institute). Data were analyzed from May 2022 to September 2023.

## Results

### Survey Samples, Response Rates, and Nonrespondents

In wave 1, 123 655 pregnant and recently pregnant people were eligible; 1500 (1.2%) were sampled. By EHR, 877 of these were unvaccinated, 551 identified as non-Hispanic Black, and 510 preferred Spanish language. In wave 2, 123 690 people were eligible and 1456 (1.2%) were sampled. By EHR, 826 of these were unvaccinated, 542 identified as non-Hispanic Black, and 476 preferred Spanish language. There were 652 respondents in wave 1 (652 of 1500 participants [43.5%]) and 575 respondents in wave 2 (575 of 1456 participants [39.5%]).

Of the 1227 total respondents, all identified as female, the mean (SD) age was 31.7 (5.6) years, 356 (29.0%) identified as Black race, 555 (45.2%) identified as Hispanic ethnicity, and 445 (36.3%) preferred the Spanish language. Eight individuals were sampled twice and completed both surveys; otherwise, respondents in wave 1 differed from respondents in wave 2. Table 1 provides EHR-derived vaccination status (unvaccinated or ≥1 doses received) and demographic information for respondents and nonrespondents by wave. Overall, people whose EHR-recorded race was Black or who were unvaccinated against COVID-19 were less likely to respond across both waves, whereas those with an EHR-recorded ethnicity of Hispanic were more likely to respond (Table 1). Respondents in wave 2 were less likely than wave 1 respondents to identify as Black (154 [26.9%] vs 202 [31.0%]; χ^2^_6_ = 13.5; *P* = .04) or non-Hispanic (295 [51.4%] vs 377 [57.8%]; χ^2^_1_ = 5.0; *P* = .03) and prefer the English language (340 [59.2%] vs 442 [67.8%]; χ^2^_1_ = 9.5; *P* = .002).

**Table 1.** Demographic Characteristics and Electronic Health Record Vaccination Status of 2956 Pregnant or Recently Pregnant People in the Vaccine Safety Datalink, Stratified by Survey Response and Wave^a^

Characteristics and levels	Wave 1^b^			Wave 2^c^			*P* value (wave 1 vs wave 2 respondents)
	Nonrespondents, No. (%) (n = 848)	Respondents, No. (%) (n = 652)	*P* value	Nonrespondents, No. (%) (n = 881)	Respondents, No. (%) (n = 575)	*P* value	
Sex							
Female	848 (100)	652 (100)	NC	881 (100)	575 (100)	NC	NC
Age group at sampling, y							
18-29	324 (38.2)	215 (33.0)	.22	358 (40.6)	215 (37.4)	.26	.27
30-39	457 (53.9)	383 (58.7)		471 (53.5)	316 (55.0)		
40-49	67 (7.9)	54 (8.3)		52 (5.9)	44 (7.7)		
Race							
American Indian or Alaska Native	1 (0.1)	7 (1.1)	<.001	3 (0.3)	11 (1.9)	<.001	.04
Asian or Pacific Islander	21 (2.5)	23 (3.5)		17 (1.9)	19 (3.3)		
Black	349 (41.2)	202 (31.0)		388 (44.0)	154 (26.9)		
White	252 (29.7)	237 (36.4)		240 (27.3)	215 (37.3)		
Multiracial	26 (3.1)	55 (8.4)		57 (6.5)	36 (6.3)		
Other^d^	20 (2.4)	84 (12.9)		5 (0.6)	73 (12.7)		
Missing/unknown	179 (21.1)	44 (6.8)		171 (19.4)	67 (11.6)		
Ethnicity							
Hispanic	346 (40.8)	275 (42.2)	<.001	297 (33.8)	280 (48.6)	<.001	.03
Non-Hispanic	478 (56.4)	377 (57.8)		584 (66.3)	295 (51.4)		
Missing or unknown	24 (2.8)	0		0	0		
Language							
English	548 (64.6)	442 (67.8)	.20	640 (72.6)	340 (59.2)	<.001	.002
Spanish	300 (35.4)	210 (32.2)		241 (27.4)	235 (40.8)		
COVID-19 vaccination status at sampling							
Unvaccinated	573 (67.6)	304 (53.4)	<.001	549 (62.4)	277 (48.1)	<.001	.63
Vaccinated (≥1 dose)	275 (32.4)	348 (46.6)		332 (37.6)	298 (51.9)		
Site							
A	101 (11.9)	66 (10.1)	.004	119 (13.5)	48 (8.3)	<.001	.07
B	62 (7.3)	61 (9.4)		92 (10.4)	35 (6.1)		
C	62 (7.3)	60 (9.2)		75 (8.5)	43 (7.5)		
D	15 (1.8)	31 (4.8)		23 (2.6)	23 (4.0)		
E	71 (8.4)	54 (8.28)		83 (9.4)	43 (7.5)		
F	216 (25.5)	158 (24.2)		198 (22.5)	182 (31.7)		
G	236 (27.8)	180 (27.6)		216 (24.5)	166 (28.9)		
H	85 (10.0)	42 (6.4)		75 (8.5)	35 (6.1)		

Abbreviation: NC, not calculated.

^a^ Vaccine Safety Datalink sites contributing data were geographically located in California, Colorado, Minnesota, Oregon, Washington, and Wisconsin.

^b^ Wave 1 of the survey was conducted from November 2021 to February 2022.

^c^ Wave 2 of the survey was conducted from October 2022 to February 2023.

^d^ Other race included individuals whose race was reported as “other” by electronic health record.

### Characteristics of Respondents by Self-Reported Vaccination Status Across Waves

Overall, 76.8% (95% CI, 71.5%-82.2%) of respondents reported receiving at least 1 COVID-19 vaccine. Of the 3 racial, ethnic, and language groups of interest, Spanish-speaking Hispanic individuals had the highest self-reported rate of any COVID-19 vaccination, 86.9% (95% CI, 82.0%-91.8%) in wave 1 and 84.2% (95% CI, 80.4%-88.1%) in wave 2; non-Hispanic Black respondents had the lowest vaccination rates, with 68.0% (95% CI, 57.9%-78.1%) in wave 1 and 69.7% (95% CI, 61.2%-78.2%) in wave 2. Of all vaccinees, 24.0% (95% CI, 15.4%-32.7%) reported 1 or more monovalent booster vaccinations in wave 1, and 25.4% (95% CI, 10.4%-40.5%) reported 1 or more Omicron booster vaccinations in wave 2 (χ^2^_1_ = 0.02; *P* = .88). Only 11.5% (95% CI, 6.3%-16.7%) of Spanish-speaking Hispanic respondents reported having received an Omicron booster vaccine in wave 2, whereas 40.2% (95% CI, 11.5%-68.9%) of Non-Hispanic White respondents self-reported 1 or more Omicron booster vaccinations in wave 2.

Table 2 compares the weighted estimates for self-reported sociodemographic characteristics in ever-vaccinated respondents and unvaccinated respondents across waves. Generally, sociodemographic characteristics did not change among vaccinees from wave 1 to wave 2, but unvaccinated individuals in wave 2 were more likely to be older, identify as White or other races, have an associate degree (or higher), and have household income above 200% of the federal poverty level (Table 2). Ever-vaccinated respondents differed from unvaccinated respondents in many respects in wave 1 (eTable 1 in Supplement 1) but only by educational attainment by wave 2 (eTable 2 in Supplement 1).

**Table 2.** Weighted Proportions of Demographic Characteristics of Pregnant or Recently Pregnant Respondents From the Vaccine Safety Datalink, Stratified by Self-Reported COVID-19 Vaccination Status Across Survey Wave 1 (November 2021 to February 2022) and Survey Wave 2 (October 2022 to February 2023)^a^

Characteristics and levels	Vaccinated with ≥1 dose (n = 886)			Unvaccinated (n = 341)		
	Wave 1 participants, % (95% CI) (n = 527)^b^	Wave 2 participants, % (95% CI) (n = 359)^c^	*P* value	Wave 1 participants, % (95% CI) (n = 125)^b^	Wave 2 participants, % (95% CI) (n = 216)^c^	*P* value
Age, y						
18-29	30.8 (21.2-40.3)	17.0 (5.7-28.7)	.09	39.8 (23.4-56.2)	30.9 (17.2-44.6)	.02
30-39	61.8 (51.8-71.8)	64.1 (48.1-80.0)		59.2 (42.8-75.6)	56.0 (40.2-71.8)	
40-49	7.4 (2.1-12.7)	18.7 (4.7-32.8)		1.0% (0-2.1)	13.1 (0.6-25.6)	
Preferred language						
English	98.5 (98.2-98.8)	98.3 (97.7-98.9)	.64	99.27 (98.9-99.6)	98.90 (98.5-99.3)	.21
Spanish	1.5 (1.2-1.8)	1.7 (1.1-2.3)		0.73 (0.4-1.1)	1.10 (0.7-1.5)	
Ethnicity^d^						
Hispanic or Latino	33.4 (23.6-43.3)	21.3 (8.2-34.3)	.17	29.9 (15.2-44.5)	28.7 (13.7-43.7)	.91
Not Hispanic or Latino	66.6 (56.7-76.4)	78.7 (65.7-91.8)		70.1 (55.5-84.8)	71.3 (56.3-86.3)	
Race^d^						
American Indian or Alaskan Native	0.02 (0-0.05)	2.4 (0-7.0)	.46	4.3 (0-11.4)	0.04 (0-0.1)	.01
Asian or Pacific Islander	12.2 (5.2-19.1)	11.2 (0.5-21.8)		8.3 (0-19.5)	0.8 (0-1.7)	
Black	6.7 (5.5-8.1)	6.8 (4.5-9.1)		10.9 (6.8-15.1)	11.1(8.0-14.3)	
Multiracial	23.0 (14.0-32.0)	22.5 (7.8-37.3)		30.8 (14.0-47.5)	28.3 (12.6-44.0)	
White	34.5 (25.1-43.9)	44.9 (28.5-61.3)		35.9 (18.8-53.0)	41.7 (26.3-57.1)	
Other^e^	17.0 (8.7-25.3)	9.1 (1.3-17.0)		2.6 (0.5-4.6)	15.3 (2.4-28.2)	
Unknown	6.5 (1.2-11.8)	3.0 (0-7.7)		7.3 (0-16.8)	2.8 (0-7.0)	
Education						
Never attended school	0.01 (0-0.04)	0.01 (0-0.03)	<.001	0	0	.01
Elementary school (or less)	0.3 (0-0.6)	0.6 (0-1.4)		0.05 (0-0.1)	0.1 (0-0.2)	
Junior high school	0.3 (0.1-0.6)	0.6 (0.1-1.2)		0.45 (0-1.3)	1.13 (0-2.5)	
High school (including GED)	27.0 (18.0-36.0)	7.9 (1.3-14.5)		41.0 (23.8-58.2)	14.3 (6.7-21.8)	
Associate or bachelor’s degree	44.3 (34.2-54.4)	64.9 (49.7-80.1)		48.5 (31.1-65.9)	64.8 (50.5-79.2)	
Master’s degree	23.4 (14.6-32.2)	18.2 (6.1-30.3)		5.4 (0-12.5)	15.5 (2.6-28.5)	
Doctorate or professional degree	2.8 (0.4-5.2)	7.3 (0-17.0)		0.02 (0-0.1)	0.9 (0-2.2)	
Rather not say	1.7 (0-4.6)	0.5 (0-1.2)		0.8 (0-1.8)	1.1 (0.1-2.2)	
Missing	0.2 (0-0.4)	0.01 (0-0.04)		3.8 (0-10.8)	2.1 (0-6.3)	
Federal poverty level, %^f^						
<100	2.9 (0.5-5.2)	1.1 (0.3-1.8)	.39	18.9 (5.2-32.6)	4.1 (1.8-6.3)	.02
101-150	3.1 (1.1-5.0)	2.9 (1.2-4.6)		1.2 (0.03-2.3)	9.9 (0.1-19.6)	
151-200	4.1 (0.1-8.1)	5.4 (0-11.9)		9.4 (0-19.1)	7.3 (0-16.3)	
>200	71.9 (62.9-80.9)	81.3 (69.8-92.8)		48.4 (31.0-65.7)	62.8 (48.6-77.0)	
Missing	18.1 (9.9-26.3)	9.3 (0-19.0)		22.1 (7.2-37.0)	16.0 (8.1-23.8)	
Site						
A	42.7 (32.1-53.2)	39.2 (21.4-56.9)	.98	31.8 (14.2-49.4)	28.5 (15.4-41.5)	.98
B	2.2 (1.2-3.3)	2.5 (0.6-4.3)		2.2 (0.7-3.6)	2.4 (0.9-3.8)	
C	5.9 (3.7-8.1)	5.8 (2.7-9.0)		4.1 (1.0-7.2)	7.9 (3.4-12.3)	
D	0.6 (0.3-1.0)	0.6 (2.7-9.0)		3.1 (1.1-5.1)	3.2 (0.9-5.4)	
E	4.3 (2.4-6.1)	4.2 (1.9-6.5)		3.6 (0.5-6.7)	4.3 (1.7-6.8)	
F	2.9 (1.9-4.0)	3.6 (1.8-5.4)		4.5 (1.7-7.3)	4.3 (2.0-6.5)	
G	35.8 (26.1-45.4)	40.1 (24.8-55.4)		47.8 (30.6-65.1)	46.4 (30.1-62.7)	
H	5.6 (2.7-8.6)	4.1 (1.8-6.4)		2.9 (0-8.3)	3.1 (1.0-5.2)	

^a^ Vaccine Safety Datalink sites contributing data were geographically located in California, Colorado, Minnesota, Oregon, Washington, and Wisconsin.

^b^ Wave 1 of the survey was conducted from November 2021 to February 2022.

^c^ Wave 2 of the survey was conducted from October 2022 to February 2023.

^d^ Self-reported race and ethnicity was used as the criterion standard; if unavailable, electronic health record data for race and/or ethnicity were used.

^e^ Other race included individuals whose race was reported as “other” by survey self-report or by electronic health record (EHR).

^f^ Estimated per 2021 Federal Poverty Level Standards, based on family size and yearly income.

### Perceptions of COVID-19 Vaccines and Vaccine Safety Across Waves

Table 3 provides weighted estimates of COVID-19 vaccine safety perceptions across waves, stratified by vaccination status. Among those reporting 1 or more COVID-19 vaccination, we observed a 34% relative decrease in the proportion in agreement that COVID-19 vaccines are safe for pregnant people and a 32% relative decrease in the proportion agreeing that COVID-19 vaccines are safe for a pregnant person’s baby (Table 3). There was a 41% relative decrease (χ^2^_1_ = 10.3; *P* < .01) in the proportion of those ever-vaccinated agreeing that most pregnant people should get a COVID-19 vaccine. Among unvaccinated respondents, attitudes toward COVID-19 vaccines were largely unfavorable and did not change across waves (Table 3). Attitudes toward COVID-19 vaccines differed by vaccination status in both waves (eTable 3 and eTable 4 in Supplement 1). In wave 1, desiring to wait until after pregnancy and perceived vaccine adverse effects were the top reported reasons for being unvaccinated (eTable 3 in Supplement 1).

**Table 3.** Weighted Estimates of Attitudes About COVID-19 and COVID-19 Vaccines Among 652 and 575 Pregnant or Recently Pregnant Persons in Wave 1 and 2, Respectively, in the Vaccine Safety Datalink, Stratified by Vaccination Status^a^^,^^b^

Attitude, belief, or intention; group; and wave	Weighted estimate, % (95% CI)			Absolute difference, %	Relative difference, %	*P* value^c^
	Negative response	Neutral response	Positive response			
COVID-19 vaccines safe for a pregnant person^d^						
Vaccinated						
Wave 1	2 (0-5)	21 (12-30)	76 (68-85)	−26	−34	<.001
Wave 2	12 (1-24)	37 (22-53)	50 (34-67)			
Unvaccinated						
Wave 1	39 (23-56)	31 (15-47)	26 (10-41)	−17	−65	.08
Wave 2	38 (23-53)	53 (37-69)	9 (3-15)			
COVID-19 vaccines safe for a pregnant person’s baby^d^						
Vaccinated						
Wave 1	6 (1-10)	20 (12-28)	74 (65-83)	−24	−32	<.001
Wave 2	15 (3-27)	34 (19-49)	51 (34-67)			
Unvaccinated						
Wave 1	40 (23-57)	34 (18-51)	22 (7-37)	−16	−71	.05
Wave 2	38 (23-53)	56 (40-71)	6 (2-11)			
Overall hesitancy about COVID-19 vaccines^e^						
Vaccinated						
Wave 1	36 (26-46)	3 (0-7)	61 (51-71)	−9	−14	.01
Wave 2	46 (30-62)	2 (1-3)	52 (36-68)			
Unvaccinated						
Wave 1	77 (63-92)	7 (0-14)	12 (0-25)	−5	−42	.96
Wave 2	79 (66-92)	14 (3-24)	7 (0-16)			
Most pregnant people should get a COVID-19 vaccine^e^						
Vaccinated						
Wave 1	10 (5-16)	15 (8-23)	74 (66-83)	−31	−41	<.001
Wave 2	36 (20-51)	20 (8-32)	44 (28-60)			
Unvaccinated						
Wave 1	62 (45-79)	28 (15-48)	6 (0-14)	−4	−1	.32
Wave 2	74 (60-87)	24 (11-38)	2 (0-4)			
If vaccinated but not boosted, will get booster^f^						
Vaccinated						
Wave 1	12 (5-19)	11 (4-18)	75 (66-85)	−30	−39	.002
Wave 2	46 (27-64)	9 (0-17)	46 (27-64)			
If unvaccinated, will get a COVID-19 vaccine^f^						
Unvaccinated						
Wave 1	67 (51-83)	17 (4-29)	17 (4-29)	−10	−58	.42
Wave 2	84 (73-97)	8 (0-18)	7 (0-16)			

^a^ Questions about COVID-19 vaccines differed from wave 1 (November 2021 to February 2022) to wave 2 (October 2022 to February 2023); in wave 1, questions were asked about “COVID-19 vaccines” (ie, original monovalent mRNA and viral vector vaccines). In wave 2, questions referred specifically to the “COVID-19 Omicron booster vaccines” (ie, bivalent booster vaccines).

^b^ Missing values were included in the calculation of the weighted proportions. Missingness accounted for between 0% and a maximum of 4% of the weighted responses.

^c^ *P* values were calculated comparing the probability of responding in the group with the most vaccine favorable attitudinal group between wave 1 and 2. *P* values were adjusted with age, education level. The absolute and relative difference and *P* values are calculated based on the difference in weighted proportion of the most vaccine favorable attitudinal group.

^d^ Response categories were “not at all/not very safe” (negative), “not sure/prefer not to answer” (neutral), and “very/somewhat safe” (positive).

^e^ Response categories were “very/somewhat hesitant” (negative), “not sure/prefer not to answer” (neutral), and “not too/not at all hesitant” (positive).

^f^ Response categories were “will probably/definitely NOT get” (negative), “not sure/prefer not to answer” (neutral), and “will probably/definitely get” (positive).

### Attitudinal Differences by Ethnicity, Race, and Preferred Language

Table 4 summarizes weighted estimates of COVID-19 vaccine safety perceptions across waves by 3 mutually exclusive ethnic, racial, and linguistic groups of interest. The greatest decreases in perceived COVID-19 vaccine safety were observed among non-Hispanic White respondents, both for themselves (relative difference, −41%; χ^2^_1_ = 5.4; *P* = .02) and their babies (relative difference, −40%; χ^2^_1_ = 3.4; *P* = .04). Hispanic Spanish-speaking respondents had the greatest decrease in the proportion of respondents “not at all” or “not too” hesitant about receiving a COVID-19 vaccine (relative difference, −42%; χ^2^_1_ = 33.9; *P* < .01), and non-Hispanic White respondents had the greatest decrease in agreement that most pregnant people should get a COVID-19 vaccine (relative difference, −43%; χ^2^_1_ = 3.5; *P* = .04). In comparison, COVID-19 vaccine attitudes did not change significantly across waves for Non-Hispanic Black respondents (Table 4).

**Table 4.** Weighted Estimates for Attitudes Regarding COVID-19 and COVID-19 Vaccines Among 652 and 575 Pregnant or Recently Pregnant Persons From Wave 1 and 2, Respectively, in the Vaccine Safety Datalink, Stratified by 3 Mutually Exclusive Ethnic, Racial, and Linguistic Groups of Interest^a^^,^^b^

Attitude, belief, or intention	Weighted estimate, % (95% CI)			Absolute difference, %	Relative difference, %	*P* value^c^
	Negative response	Neutral response	Positive response			
COVID-19 vaccines safe for pregnant person^d^						
Non-Hispanic Black						
Wave 1	20 (11-29)	28 (19-37)	50 (40-61)	−5	−10	.30
Wave 2	16 (9-23)	39 (29-48)	45 (35-56)			
Hispanic, any race, Spanish						
Wave 1	4 (1-7)	19 (14-25)	76 (70-82)	−24	−31	.002
Wave 2	4 (1-6)	44 (37-51)	53 (46-60)			
Non-Hispanic White						
Wave 1	16 (3-29)	11 (0-23)	72 (56-88)	−30	−41	.02
Wave 2	23 (4-42)	35 (13-56)	43 (19-66)			
COVID-19 vaccines safe for pregnant person’s baby^d^						
Non-Hispanic Black						
Wave 1	24 (14-33)	32 (23-42)	43 (33-53)	−4	−10	.35
Wave 2	20 (13-28)	41 (31-51)	39 (29-49)			
Hispanic, any race, Spanish						
Wave 1	8 (4-11)	23 (17-29)	68 (62-75)	−25	−36	.006
Wave 2	7 (3-11)	49 (42-56)	44 (37-51)			
Non-Hispanic White						
Wave 1	17 (4-29)	17 (3-30)	67 (50-83)	−27	−40	.04
Wave 2	22 (3-41)	38 (16-59)	40 (17-64)			
Overall hesitancy about COVID-19 vaccines^e^						
Non-Hispanic Black						
Wave 1	55 (45-65)	6 (1-11)	38 (28-47)	−14	−37	.69
Wave 2	50 (39-60)	16 (8-23)	34 (24-44)			
Hispanic, any race, Spanish						
Wave 1	32 (25-39)	5 (2-8)	62 (56-69)	−26	−42	<.001
Wave 2	33 (27-40)	30 (24-37)	36 (29-44)			
Non-Hispanic White						
Wave 1	38 (21-55)	0 (0-1)	62 (45-79)	−17	−28	.10
Wave 2	54 (31-78)	1 (0-2)	45 (21-68)			
Most pregnant people should get a COVID-19 vaccine^f^						
Non-Hispanic Black						
Wave 1	25 (16-35)	31 (22-42)	43 (33-53)	−10	−24	.20
Wave 2	32 (23-41)	36 (26-45)	32 (23-43)			
Hispanic, any race, Spanish						
Wave 1	6 (2-9)	12 (7-17)	82 (76-87)	−25	−31	<.001
Wave 2	8 (4-11)	36 (29-43)	57 (50-64)			
Non-Hispanic White						
Wave 1	21 (8-34)	15 (1-28)	64 (47-80)	−28	−43	.04
Wave 2	46 (23-69)	17 (4-31)	36 (13-60)			
If unvaccinated, will get a COVID-19 vaccine^g^						
Non-Hispanic Black						
Wave 1	52 (32-73)	28 (9-46)	20 (3-37)	−4	−22	.92
Wave 2	84 (73-95)	0 (0-1)	16 (5-27)			
Hispanic, any race, Spanish						
Wave 1	21 (4-38)	26 (8-44)	53 (33-74)	−28	−53	.02
Wave 2	45 (33-57)	30 (19-41)	25 (15-35)			
Non-Hispanic White						
Wave 1	83 (57-100)	15 (0-41)	2 (0-4)	13	8	.05
Wave 2	83 (57-100)	2 (0-5)	15 (0-41)			
If vaccinated but not boosted, will get a booster^g^						
Non-Hispanic Black						
Wave 1	23 (11-35)	19 (8-30)	58 (45-71)	−20	−35	.03
Wave 2	49 (35-64)	13 (3-22)	38 (24-52)			
Hispanic, any race, Spanish						
Wave 1	1 (0-4)	16 (9-24)	79 (71-88)	−11	−13	.02
Wave 2	14 (8-20)	17 (11-24)	69 (61-77)			
Non-Hispanic White						
Wave 1	4 (0-9)	11 (0-28)	83 (65-100)	−52	−63	.02
Wave 2	68 (44-93)	1 (0-3)	31 (7-55)			

^a^ Questions about COVID-19 vaccines differed from wave 1 (November 2021 to February 2022) to wave 2 (October 2022 to February 2023); in wave 1, questions were asked about “COVID-19 vaccines” (ie, original monovalent mRNA and viral vector vaccines). In wave 2, questions referred specifically to the “COVID-19 Omicron booster vaccines” (ie, bivalent booster vaccines).

^b^ Missing values were included in the calculation of the weighted proportions. Missingness accounted for between 0% and a maximum of 4% of the weighted responses.

^c^ *P* values were calculated comparing the probability of responding in the group with the most vaccine favorable attitudinal group between wave 1 and 2. *P* values were adjusted with age, education level. The absolute and relative difference and *P* values are calculated based on the difference in weighted proportion of the most vaccine favorable attitudinal group.

^d^ Response categories were “not very/not at all safe” (negative), “not sure/prefer not to answer” (neutral), and “very/somewhat safe” (positive).

^e^ Response categories were “very/somewhat hesitant” (negative), “not sure/prefer not to answer” (neutral), and “not too/not at all hesitant” (positive).

^f^ Response categories were “disagree/strongly disagree” (negative), “not sure/prefer not to answer” (neutral), and “agree/strongly agree” (positive).

^g^ Response categories were “will probably/definitely NOT get” (negative), “not sure/prefer not to answer” (neutral), and “will probably/definitely get” (positive).

### Trust in the CDC and Health Care Practitioners

In the second survey wave, respondents’ most trusted sources for information about COVID-19 and COVID-19 vaccines varied by vaccination status (eTable 4 in Supplement 1). Among ever-vaccinated participants, 34.4% (95% CI, 18.5%-50.3%) trusted the CDC the most for this information, and a similar proportion, 34.4% (95% CI, 18.5%-50.2%), chose their physician. Of unvaccinated participants, 9.2% (95% CI, 0%-19.0%) reported trusting the CDC most, with 11.0% (95% CI, 4.5%-17.5%) reporting their physician. Among all Non-Hispanic Black respondents, 32.5% (95% CI, 22.6%-42.4%) reported trusting the CDC most and 18.7% (95% CI, 11.1%-26.3%) their physicians. Conversely, Spanish-speaking Hispanic participants had a lower proportion trusting the CDC (26.1%; 95% CI, 19.6%-32.6%), but a higher proportion trusting their physicians (35.1%; 95% CI, 28.1%-42.0%). Among Non-Hispanic White participants, 26.6% (95% CI, 5.7%-47.6%) trusted the CDC most while 38.2% (95% CI, 14.5%-62.0%) trusted their physicians most.

## Discussion

Among diverse pregnant and recently pregnant VSD members during the latter part of the COVID-19 public health emergency, we observed significant changes in perceptions of COVID-19 vaccine safety over time. First, we observed a significant change over time in respondent groups’ perceptions of COVID-19 vaccine safety for pregnant persons and their infants. While we did not survey the same individuals at 2 time points, the general trends we observed among those who had received at least 1 COVID-19 vaccine and among racially, ethnically, and linguistically diverse groups are concerning. Substantial evidence continues to accrue supporting COVID-19 vaccine safety for pregnant people,^10,50,51,52^ but the concomitant spread of misinformation^53,54^ may partially explain our declines in perceived safety. A Kaiser Family Foundation survey ending in June 2023 found that 27% of respondents believed COVID-19 vaccines have been shown to cause infertility; 68% of respondents were uncertain whether the claim was definitely true or definitely false.^55^ Interestingly, 94% of adults in the Kaiser survey reported “a great deal” or “fair amount” of trust in their physician to make the appropriate vaccination recommendations for them, with the next most highly trusted information source being the CDC.^55^ Our data suggest more modest levels of trust in the CDC and physicians with notable differences by race, ethnicity, and language preference. Future work could study perceived safety of COVID-19 vaccines as a function of message tailoring for diverse groups.

Weighted rates of receipt of 1 or more COVID-19 vaccines were highest for Spanish-speaking Hispanic respondents in both waves. This finding contrasts with early and late pandemic studies identifying low COVID-19 vaccination rates and delayed first doses among Hispanic individuals.^16,17,18,19,21,56,57,58^ The cultural phenomenon of *respeto*, by which Spanish-speaking families have been observed to subjugate personal concerns to clinical expertise,^59^ may explain this early difference. However, by wave 2, Spanish-speaking Hispanic respondents became less confident in COVID-19 vaccine safety, were less in agreement that pregnant people should get a COVID-19 vaccine, and had significantly lower rates of Omicron booster vaccine receipt. Culturally tailored COVID-19 vaccine interventions for Spanish-speaking Hispanic communities exist^60^ and should be carefully implemented in diverse contexts. We suggest they engage health care practitioners, which were most highly trusted for information by Spanish speaking Hispanic participants in our survey.

Perceptions in non-Hispanic Black and non-Hispanic White respondents are also concerning. Non-Hispanic Black respondents were most concerned about safety of COVID-19 vaccines in both waves. Meanwhile, Non-Hispanic White respondents became significantly more hesitant about COVID-19 vaccines, less sure of COVID-19 vaccine safety, and more uncertain that pregnant people should get a COVID-19 vaccine. These trends in non-Hispanic White individuals, who were early adopters of COVID-19 vaccines,^16,17,19,20^ are an important finding and deserve attention. Persistently more unfavorable perceptions of COVID-19 vaccine safety in Black respondents underline the importance of continually attending to safety concerns in historically marginalized communities.

### Limitations

This study has limitations. Survey nonresponse may have influenced our results, as nonrespondents differed from respondents, respondents differed across waves, response rates decreased as the pandemic evolved (as others have noted), and increasing nonresponse may itself indicate decreasing confidence in COVID-19 vaccines.^61,62^ Second, we sampled Spanish-speaking Hispanic members at only 2 VSD sites; findings may not be generalizable to Spanish-speaking Hispanic individuals in other areas. Third, we had low numbers of respondents from non-Hispanic Asian, Pacific Islander, and Indigenous groups, which caused significant uncertainty in their attitudinal estimates; studies have shown the highest COVID-19 vaccination rates in individuals who identify as non-Hispanic Asian.^20,54^ Question wording about COVID-19 vaccines and COVID booster vaccines differed between survey waves due to changing federal recommendations. Additionally, we did not ask respondents if they were pregnant at the time of survey completion, which could have provided additional insights.

## Conclusions

During the latter portion of the public health emergency, we observed significant changes in COVID-19 vaccine hesitancy and perceptions of COVID-19 vaccine safety among insured pregnant and recently pregnant people. These differences, despite accruing evidence of COVID-19 vaccine safety in this high-risk group, are concerning for clinicians and public health officials.

## Notes

Supplement 1. **eTable 1.** Weighted Demographic Characteristics of 652 Pregnant or Recently Pregnant Respondents From the Vaccine Safety Datalink, Stratified by Self-Reported COVID-19 Vaccination Status, November 2021 to February 2022 (Wave 1) **eTable 2.** Weighted Demographic Characteristics of 575 Pregnant or Postpartum Respondents From the Vaccine Safety Datalink, Stratified by Self-Reported COVID-19 Vaccination Status, October 2022 to February 2023 (Wave 2) **eTable 3.** Weighted Differences in Attitudes Toward COVID-19 Vaccines Among 652 Pregnant or Postpartum Vaccine Safety Datalink Members, Stratified by Vaccination Status, November 2021 to February 2022 (Wave 1) **eTable 4.** Weighted Differences in Attitudes About COVID-19 Vaccines Among 575 Pregnant or Recently Pregnant People in the Vaccine Safety Datalink, Stratified by Vaccination Status, October 2022 to February 2023 (Wave 2)

Supplement 2.
Data Sharing Statement
