# Supplementary material for: Attitudes Toward COVID-19 Vaccines Among Pregnant and Recently Pregnant Individuals
Source: JAMA Netw Open. 2024 Apr 8;7(4):e245479. doi: 10.1001/jamanetworkopen.2024.5479 (PMC11002697; doi:10.1001/jamanetworkopen.2024.5479)
Supplement: Supplement 1. — eTable 1. Weighted Demographic Characteristics of 652 Pregnant or Recently Pregnant Respondents From the Vaccine Safety Datalink, Stratified by Self-Reported COVID-19 Vaccination Status, November 2021 to February 2022 (Wave 1) eTable 2. Weighted Demographic Characteristics of 575 Pregnant or Postpartum Respondents From the Vaccine Safety Datalink, Stratified by Self-Reported COVID-19 Vaccination Status, October 2022 to February 2023 (Wave 2) eTable 3. Weighted Differences in Attitudes Toward COVID-19 Vaccines Among 652 Pregnant or Postpartum Vaccine Safety Datalink Members, Stratified by Vaccination Status, November 2021 to February 2022 (Wave 1) eTable 4. Weighted Differences in Attitudes About COVID-19 Vaccines Among 575 Pregnant or Recently Pregnant People in the Vaccine Safety Datalink, Stratified by Vaccination Status, October 2022 to February 2023 (Wave 2) [file jamanetwopen-e245479-s001.pdf]

Supplemental Online Content

Williams JTB, Kurlandsky K, Breslin K, et al. Changing attitudes toward COVID-19 vaccines among pregnant and recently pregnant individuals. *JAMA Netw Open*. 2024;7(4):e245479
doi:10.1001/jamanetworkopen.2024.5479

eTable 1. Weighted Demographic Characteristics of 652 Pregnant or Recently Pregnant Respondents From the Vaccine Safety Datalink, Stratified by Self-Reported COVID-19 Vaccination Status, November 2021 to February 2022 (Wave 1)

eTable 2. Weighted Demographic Characteristics of 575 Pregnant or Postpartum Respondents From the Vaccine Safety Datalink, Stratified by Self-Reported COVID-19 Vaccination Status, October 2022 to February 2023 (Wave 2)

eTable 3. Weighted Differences in Attitudes Toward COVID-19 Vaccines Among 652 Pregnant or Postpartum Vaccine Safety Datalink Members, Stratified by Vaccination Status, November 2021 to February 2022 (Wave 1)

eTable 4. Weighted Differences in Attitudes About COVID-19 Vaccines Among 575 Pregnant or Recently Pregnant People in the Vaccine Safety Datalink, Stratified by Vaccination Status, October 2022 to February 2023 (Wave 2)

This supplemental material has been provided by the authors to give readers additional information about their work.

© 2024 Williams JTB et al. *JAMA Netw Open.*

eTable 1. Weighted Demographic Characteristics of 652 Pregnant or Recently Pregnant Respondents From the Vaccine Safety Datalink, Stratified by Self-Reported COVID-19 Vaccination Status, November 2021 to February 2022 (Wave 1)

Characteristics and Levels	Vaccinated (≥ 1 dose) % (95% CI), N = 527	Unvaccinated % (95% CI), N = 125	p-value
Age[a]			0.12
18-29 years	30.8% (21.2%, 40.3%)	39.8% (23.5%, 56.2%)	
30-39 years	61.8% (51.8%, 71.9%)	59.2% (42.8%, 75.6%)	
40-49 years	7.4% (2.1%, 12.7%)	1.0% (0.0%, 2.1%)	
Preferred Language			0.01
English	98.5% (98.2%, 98.8%)	99.3% (98.9%, 99.6%)	
Spanish	1.5% (1.2%, 1.8%)	0.7% (0.4%, 1.1%)	
Ethnicity[b]			0.70
Hispanic or Latino	33.5% (23.6%, 43.3%)	29.9% (14.7%, 45.0%)	
Not Hispanic or Latino	66.5% (56.7%, 76.4%)	70.1% (55.0%, 85.3%)	
Race[b]			0.03
Am. Indian or Alaskan Native	0.02% (0.0%, 0.05%)	4.3% (0.0%, 11.3%)	
Asian or Pacific Islander	12.2% (5.2%, 19.2%)	8.3% (0.0%, 19.4%)	
Black	6.8% (5.6%, 7.9%)	10.9% (6.3%, 15.6%)	
Multiracial	23.0% (14.0%, 32.7%)	30.8% (14.2%, 47.3%)	
White	34.5% (25.1%, 43.9%)	35.9% (19.0%, 52.8%)	
Other	17.0% (8.7%, 25.3%)	2.5% (0.4%, 4.7%)	
Unknown	6.5 (1.2, 11.8%)	7.3% (0.0%, 16.8%)	
Education			<0.001
Never attended school	0.01% (0.0%, 0.04%)	0.0% (NC)	
Elementary school (or less)	0.28% (0.0%, 0.6%)	0.05% (0.0%, 0.1%)	
Junior high school	0.35% (0.1%, 0.6%)	0.4% (0.0%, 1.3%)	
High school (including GED)	27.0% (18.0%, 36.0%)	41.0% (24.1%, 58.0%)	
College graduate	44.3% (34.2%, 54.4%)	48.5% (31.2%, 65.7%)	
Advanced (e.g., MPH, JD)	26.2% (17.2%, 35.2%)	5.4% (0.0%, 12.5%)	
Not Sure/PNTA	1.7% (0.0%, 4.5%)	0.8% (0.0%, 1.9%)	
Missing	0.2% (0.0%, 0.4%)	3.8% (0.0%, 10.7%)	
Income			0.003
$0 - $25,000	3.0% (0.6%, 5.4%)	18.9% (5.3%, 32.6%)	
$25,001 - $50,000	10.9% (5.0%, 16.8%)	16.6% (3.8%, 29.4%)	
$50,001 - $75,000	20.6% (12.0%, 29.2%)	14.4% (2.9%, 25.9%)	
$75,001 - $100,000	11.9% (5.1%, 18.6%)	13.4% (1.3%, 25.5%)	
$100,001 or more	38.7% (28.8%, 48.7%)	19.3% (5.3%, 33.3%)	
Not Sure/PNTA	14.7% (7.2%, 22.2%)	13.6% (2.2%, 24.9%)	
Missing	0.2% (0.1%, 0.4%)	3.8% (0%, 10.8%)	
Family Size			0.85
1	5.6% (1.0%, 10.1%)	8.0% (0%, 17.6%)	
2	28.0% (18.8%, 37.3%)	26.2% (10.7%, 41.8%)	
3	30.9% (21.4%, 40.5%)	24.5% (10.2%, 38.7%)	
4	13.7% (7.1%, 20.2%)	14.6% (2.5%, 26.7%)	
5	8.1% (2.4%, 13.7%)	6.9% (0.0%, 16.0%)	
6	8.5% (2.9%, 14.1%)	8.6% (0.0%, 18.2%)	
7 or more	1.4% (0.0%, 3.6%)	0.7% (0.0%, 1.7%)	
Not Sure/PNTA	3.3% (0.0%, 7.3%)	6.6% (0.0%, 15.7%)	
Missing	0.5% (0.04%, 0.9%)	3.8% (0.0%, 10.7%)	

© 2024 Williams JTB et al. *JAMA Netw Open.*

Characteristics and Levels	Vaccinated (≥ 1 dose) % (95% CI), N = 527	Unvaccinated % (95% CI), N = 125	p-value
Federal Poverty Level[c]			<0.001
<100%	2.9% (0.5%, 5.2%)	18.9% (5.3%, 32.6%)	
101-150%	3.1% (1.1%, 5.0%)	1.2% (0.01%, 2.4%)	
151-200%	4.1% (0.1%, 8.1%)	9.4% (0%, 19.1%)	
>200%	71.9% (62.9%, 80.9%)	48.4% (31.2%, 65.6%)	
Not Sure/PNTA	17.7% (9.5%, 25.9%)	18.3% (4.7%, 31.9%)	
Missing	0.4% (0%, 0.8%)	3.8% (0%, 10.7%)	

Abbreviations: PNTA, prefer to not answer; NC, not calculated.

[a] At time of sampling (August 2021).

[b] Self-reported race and ethnicity were the criterion standard and only supplemented by EHR race and ethnicity for individuals who did not self-report race and ethnicity on survey.

[c] Estimated per 2021 Federal Poverty Level Standards, based on family size and yearly income.

© 2024 Williams JTB et al. *JAMA Netw Open.*

eTable 2. Weighted Demographic Characteristics of 575 Pregnant or Postpartum Respondents From the Vaccine Safety Datalink, Stratified by Self-Reported COVID-19 Vaccination Status, October 2022 to February 2023 (Wave 2)

Characteristics and Levels	Vaccinated (≥ 1 dose) % (95% CI), N = 359	Unvaccinated % (95% CI), N = 216	*P*
Age[a]			0.35
18-29 years	17.2% (5.6%, 28.8%)	30.9% (17.3%, 44.5%)	
30-39 years	64.1% (48.0%, 80.1%)	56.0% (40.4%, 71.7%)	
40-59 years	18.7% (4.6%, 32.9%)	13.1% (0.7%, 25.4%)	
Preferred Language			0.15
English	98.3% (97.6%, 98.8%)	98.9% (98.4%, 99.3%)	
Spanish	1.7% (1.2%, 2.3%)	1.1% (0.7%, 1.5%)	
Ethnicity[b]			0.46
Hispanic or Latino	21.3% (8.2%, 34.4%)	28.7% (13.7%, 43.6%)	
Not Hispanic or Latino	78.7% (65.6%, 91.8%)	71.3% (56.4%, 86.3%)	
Race[b]			0.26
Am. Indian or Alaskan Native	2.4% (0.0%, 7.0%)	0.04% (0.0%, 0.1%)	
Asian or Pacific Islander	11.2% (0.4%, 21.9%)	0.8% (0.0%, 1.7%)	
Black	6.8% (4.8%, 8.8%)	11.1% (7.6%, 14.6%)	
Multiracial	22.5% (7.7%, 37.4%)	28.3% (12.6%, 44.0%)	
White	44.9% (28.4%, 61.4%)	41.7% (26.3%, 57.0%)	
Other	9.1% (1.3%, 16.9%)	15.3% (2.5%, 28.1%)	
Unknown	3.0% (0%, 7.7%)	2.8% (0%, 6.9%)	
Education			<0.001
Never attended school	0.01% (0.0%, 0.03%)	0.0% (NC)	
Elementary school (or less)	0.4% (0.0%, 1.4%)	0.1% (0.03%, 0.2%)	
Junior high school	0.6% (0.1%, 1.2%)	1.1% (0.0%, 2.5%)	
High school (including GED)	7.9% (1.3%, 14.5%)	14.3% (6.4%, 22.2%)	
Associate or bachelor's degree	64.9% (49.7%, 80.2%)	64.8% (50.5%, 79.1%)	
Master's degree	18.2% (6.0%, 30.4%)	15.5% (2.8%, 28.3%)	
Doctorate/Professional degree	7.3% (0.0%, 17.1%)	0.9% (0.0%, 2.2%)	
Rather not say	0.4% (0.0%, 1.2%)	1.1% (0.1%, 2.2%)	
Missing	0.01% (0.0%, 0.04%)	2.1% (0.0%, 6.3%)	
Income			0.14
No income	0.7% (0.0%, 1.6%)	2.7% (0.0%, 6.9%)	
$1 - $25,000	1.0% (0.3%, 1.8%)	3.7% (1.5%, 5.9%)	
$25,001 - $50,000	15.1% (3.6%, 26.6%)	31.1% (15.5%, 46.7%)	
$50,001 - $75,000	7.7% (1.1%, 14.4%)	6.6% (3.5%, 9.7%)	
$75,001 - $100,000	15.0% (3.3%, 26.7%)	7.6% (1.4%, 13.8%)	
$100,001 or more	51.8% (35.3%, 68.2%)	35.4% (19.2%, 51.6%)	
Not Sure	2.0% (0.9%, 3.2%)	3.4% (1.3%, 5.5%)	
Rather Not Say	6.5% (0.0%, 16.3%)	9.5% (3.1%, 16.0%)	
Missing	0.01% (0.0%, 0.02%)	0.0% (NC)	
Family Size			0.57
1	8.2% (0.0%, 17.9%)	10.8% (0.5%, 21.2%)	
2	27.2% (12.3%, 42.1%)	13.3% (1.4%, 25.2%)	
3	29.2% (13.9%, 44.6%)	24.6% (10.4%, 38.7%)	
4	19.0% (6.8%, 31.3%)	24.0% (11.9%, 36.1%)	
Family Size (continued)			0.85

© 2024 Williams JTB et al. *JAMA Netw Open.*

Characteristics and Levels	Vaccinated (≥ 1 dose) % (95% CI), N = 359	Unvaccinated % (95% CI), N = 216	*P*
5	12.1% (1.1%, 23.0%)	18.4% (5.1%, 31.7%)	
6	0.8% (0.1%, 1.5%)	4.3% (0.0%, 8.7%)	
7 or more	2.4% (0.0%, 7.2%)	2.4% (0.5%, 4.2%)	
Missing	0.9% (0.04%, 1.8%)	2.4% (0.6%, 4.1%)	
Federal Poverty Level[c]			0.12
<100%	1.0% (0.3%, 1.8%)	4.1% (1.8%, 6.4%)	
101-150%	2.9% (1.3%, 4.5%)	9.9% (0.1%, 19.6%)	
151-200%	5.4% (0.0%, 11.9%)	7.3% (0.0%, 16.3%)	
>200%	81.3% (69.7%, 92.9%)	62.8% (48.6%, 77.0%)	
Missing	9.3% (0%, 19.0%)	16.0% (8.0%, 23.9%)	

Abbreviations: NC, not calculated

[a] At time of sampling (November 2022).

[b] Self-reported race and ethnicity were the criterion standard and only supplemented by EHR race and ethnicity for individuals who did not self-report race and ethnicity on survey.

[c] Estimated per 2021 Federal Poverty Level Standards, based on family size and yearly income.

© 2024 Williams JTB et al. *JAMA Netw Open.*

eTable 3. Weighted Differences in Attitudes Toward COVID-19 Vaccines Among 652 Pregnant or Postpartum Vaccine Safety Datalink Members, Stratified by Vaccination Status, November 2021 to February 2022 (Wave 1)

Attitudes, Knowledge, Beliefs	Vaccinated (≥1 dose) % (95% CI), N = 527	Unvaccinated % (95% CI), N = 125	*P*
If vaccinated but not boosted, will get a booster			NA
Will probably/definitely get	57.3% (47.2%, 67.3%)	NA	
Will probably/definitely NOT get	9.1% (3.3%, 14.9%)	NA	
Not sure/PNTA	8.2% (2.8%, 13.6%)	NA	
Missing	25.4% (16.6%, 34.3%)	NA	
If unvaccinated, will get a COVID-19 vaccine			NA
Will probably/definitely	NA	16.6% (3.0%, 30.2%)	
Will probably/definitely NOT	NA	66.9% (49.7%, 84.1%)	
Not sure/PNTA	NA	16.5% (3.0%, 30.1%)	
Missing	NA	0.0% (NC)	
If vaccinated, MOST important reason			NA
COVID-19 can cause a serious illness	24.7% (15.6%, 33.7%)	NA	
I am an essential worker	8.8% (3.5%, 14.1%)	NA	
To protect myself and my baby	39.5% (29.5%, 49.4%)	NA	
To protect others in my community	3.6% (0.4%, 6.8%)	NA	
Workplace or school requirement	8.2% (2.2%, 14.1%)	NA	
Other	14.6% (7.2%, 22.1%)	NA	
Not sure/PNTA	0.5% (0.0%, 1.0%)	NA	
Missing	0.2% (0%, 0.6%)	NA	
If unvaccinated, MOST important reason			NA
Will wait until pregnancy over	NA	34.9% (17.2%, 52.5%)	
Concerned about side effects	NA	28.8% (12.9%, 44.8%)	
Don't trust development process	NA	6.7% (4.2%, 9.2%)	
Don't think vaccines are effective	NA	2.6% (0.4%, 4.8%)	
Other	NA	20.1% (4.8%, 35.5%)	
Not sure/PNTA	NA	6.9% (0.0%, 14.3%)	
Missing	NA	0.0% (NC)	
COVID-19 vaccines safe for pregnant person			<0.001
Very/somewhat safe	76.5% (67.6%, 85.4%)	25.6% (10.3%, 40.9%)	
Not very/not at all safe	2.5% (0.1%, 4.8%)	39.2% (22.6%, 55.8%)	
Not sure/PNTA	21.0% (12.3%, 29.7%)	31.3% (15.2%, 47.4%)	
Missing	0.1% (0.0%, 0.2%)	3.9% (0.0%, 10.8%)	
COVID-19 vaccines safe for pregnant person's baby			<0.001
Very/somewhat safe	74.3% (65.4%, 83.2%)	21.8% (6.9%, 36.6%)	
Not very/not at all safe	5.6% (1.3%, 10.0%)	39.9% (23.3%, 56.5%)	
Not sure/PNTA	20.0% (11.7%, 28.2%)	34.4% (18.0%, 50.8%)	
Missing	0.1% (0.0%, 0.2%)	3.9% (0.0%, 10.8%)	
Overall hesitancy about COVID-19 vaccines			<0.001
Very/somewhat hesitant	35.9% (24.0%, 45.8%)	77.2% (62.5%, 91.9%)	
Not too/not at all hesitant	61.0% (50.9%, 71.1%)	12.3% (0.1%, 24.4%)	
Not sure/PNTA	3.0% (0.0%, 6.9%)	6.8% (0.0%, 14.1%)	
Missing	0.1% (0.0%, 0.2%)	3.8% (0.0%, 10.7%)	

Abbreviations: NA, not applicable.; NC, not calculated; PNTA, prefer not to answer.

© 2024 Williams JTB et al. *JAMA Netw Open.*

eTable 4. Weighted Differences in Attitudes About COVID-19 Vaccines Among 575 Pregnant or Recently Pregnant People in the Vaccine Safety Datalink, Stratified by Vaccination Status, October 2022 to February 2023 (Wave 2)

Attitudes, Knowledge, Beliefs	Vaccinated (≥ 1 dose) % (95% CI), N = 359	Unvaccinated % (95% CI), N = 216	*P*
If not Omicron boosted, will get an Omicron booster[a]			<0.001
Will probably/definitely get	45.7% (27.3%, 64.2%)	2.1% (0.8%, 3.5%)	
Will probably/definitely NOT get	45.7% (27.2%, 64.2%)	84.1% (71.7%, 96.6%)	
Not Sure/Rather Not Say	8.6% (0.0%, 17.3%)	13.1% (0.7%, 25.6%)	
Missing	0.0% (NC)	0.6% (0.0%, 1.9%)	
If unvaccinated, will get a COVID-19 vaccine			NA
Will probably/definitely get	NA	7.0% (0.0%, 16.0%)	
Will probably/definitely NOT get	NA	84.8% (72.1%, 97.4%)	
Not Sure/Rather Not Say	NA	8.3% (0.0%, 18.2%)	
Omicron booster vaccines safe for pregnant person			<0.001
Very/somewhat safe	50.4% (33.9%, 66.9%)	8.9% (2.7%, 15.1%)	
Not very/not at all safe	12.4% (0.9%, 23.9%)	38.0% (22.7%, 53.3%)	
Not Sure/Rather Not Say	37.2% (21.5%, 52.8%)	53.1% (37.3%, 68.9%)	
Omicron booster vaccines safe for pregnant person's baby			<0.001
Very/somewhat safe	50.5% (34.0%, 67.0%)	6.3% (1.6%, 11.0%)	
Not very/not at all safe	15.2% (3.1%, 27.3%)	37.9% (22.6%, 53.3%)	
Not Sure/Rather Not Say	34.3% (18.9%, 49.6%)	55.8% (40.2%, 71.4%)	
Overall hesitancy about Omicron booster vaccines			<0.001
Very/somewhat hesitant	46.0% (30.0%, 61.9%)	78.9% (65.9%, 91.9%)	
Not too/not at all hesitant	52.3% (36.3%, 68.3%)	7.1% (0.0%, 16.0%)	
Not sure/Rather Not Say	1.7% (0.8%, 2.6%)	13.7% (3.2%, 24.3%)	
Missing	0.0% (NC)	0.3% (0.0%, 0.7%)	
Most important source for information			<0.001
Centers for Disease Control and Prevention (CDC)	34.4% (18.5%, 50.3%)	9.2% (0.0%, 18.9%)	
Family and/or friends	4.0% (0.0%, 8.7%)	5.4% (0.6%, 10.2%)	
My doctor or healthcare provider	34.4% (18.5%, 50.2%)	11.0% (4.5%, 17.6%)	
News sources	0.2% (0.0%, 0.5%)	7.3% (0.0%, 16.9%)	
State or local health Departments	0.7% (0.0%, 1.6%)	0.6% (0.0%, 1.4%)	
I don't trust anyone	9.4% (1.4%, 17.4%)	24.2% (9.7%, 38.7%)	
Other	3.3% (0.0%, 8.1%)	11.3% (0.8%, 21.7%)	
Not sure	2.7% (1.0%, 4.3%)	18.9% (5.9%, 31.8%)	
Rather Not Say	8.3% (0.0%, 18.9%)	12.1% (2.2%, 22.0%)	
Unknown	2.7% (0.0%, 7.3%)	0.1% (0.0%, 0.2%)	

Abbreviations: NC, not calculated; NA, not applicable

[a]Unvaccinated for this comparison is equivalent to those who reported they had NOT yet received a COVID-19 Omicron (a.k.a. bivalent) booster vaccine; n = 508 for the comparison, excluding those reporting being boosted.

© 2024 Williams JTB et al. *JAMA Netw Open.*
