# Supplementary material for: Attitudes Toward COVID-19 Vaccines Among Pregnant and Recently Pregnant Individuals
Source: JAMA Netw Open. 2024 Apr 8;7(4):e245479. doi: 10.1001/jamanetworkopen.2024.5479 (PMC11002697; doi:10.1001/jamanetworkopen.2024.5479)
Supplement: Supplement 2. — Data Sharing Statement [file jamanetwopen-e245479-s002.pdf]

Data Sharing Statement

Williams. Attitudes Toward COVID-19 Vaccines Among Pregnant and Recently Pregnant Individuals. *JAMA Netw Open*. Published April 08, 2024.
doi:10.1001/jamanetworkopen.2024.5479

Data
Data available: Yes
Data types: Other (please specify), Deidentified participant data, Data dictionary
Additional Information: Survey forms.
How to access data: Data is available upon request through the Centers for Disease Control and Prevention published acquisition guidelines.
When available: With publication

Supporting Documents
Document types: Other (please specify)
Additional Information: Survey forms.
How to access documents: Data is available upon request.
When available: With publication

Additional Information
Who can access the data: Researchers whose proposed use of the data has been approved by the CDC.
Types of analyses: A specified purpose as approved by the CDC.
Mechanisms of data availability: With a signed data access agreement per CDC requirements.
